# Gene Expression and Proteome Analysis as Sources of Biomarkers in Basal Cell Carcinoma

**DOI:** 10.1155/2016/9831237

**Published:** 2016-08-08

**Authors:** Mihai Lupu, Constantin Caruntu, Mihaela Adriana Ghita, Vlad Voiculescu, Suzana Voiculescu, Adrian E. Rosca, Ana Caruntu, Liliana Moraru, Iris Maria Popa, Bogdan Calenic, Maria Greabu, Daniela Elena Costea

**Affiliations:** ^1^Department of Dermatology and Allergology, Elias Emergency University Hospital, 011461 Bucharest, Romania; ^2^Department of Physiology, “Carol Davila” University of Medicine and Pharmacy, 050474 Bucharest, Romania; ^3^Department of Dermatology, “Prof. N. C. Paulescu” National Institute of Diabetes, Nutrition and Metabolic Diseases, 020475 Bucharest, Romania; ^4^Dermatology Research Laboratory, “Carol Davila” University of Medicine and Pharmacy, 050474 Bucharest, Romania; ^5^Department of Oral and Maxillofacial Surgery, “Carol Davila” Central Military Emergency Hospital, 010825 Bucharest, Romania; ^6^Department of Plastic and Reconstructive Surgery, “Bagdasar Arseni” Clinical Emergency Hospital, 041915 Bucharest, Romania; ^7^Department of Biochemistry, Faculty of Dental Medicine, University of Medicine and Pharmacy “Carol Davila”, 050474 Bucharest, Romania; ^8^Gade Laboratory for Pathology and Centre for Cancer Biomarkers (CCBio), Department of Clinical Medicine, University of Bergen, 5021 Bergen, Norway; ^9^Department of Pathology, Haukeland University Hospital, 5021 Bergen, Norway

## Abstract

Basal cell carcinoma (BCC) is the world's leading skin cancer in terms of frequency at the moment and its incidence continues to rise each year, leading to profound negative psychosocial and economic consequences. UV exposure is the most important environmental factor in the development of BCC in genetically predisposed individuals, this being reflected by the anatomical distribution of lesions mainly on sun-exposed skin areas. Early diagnosis and prompt management are of crucial importance in order to prevent local tissue destruction and subsequent disfigurement. Although various noninvasive or minimal invasive techniques have demonstrated their utility in increasing diagnostic accuracy of BCC and progress has been made in its treatment options, recurrent, aggressive, and metastatic variants of BCC still pose significant challenge for the healthcare system. Analysis of gene expression and proteomic profiling of tumor cells and of tumoral microenvironment in various tissues strongly suggests that certain molecules involved in skin cancer pathogenic pathways might represent novel predictive and prognostic biomarkers in BCC.

## 1. Introduction 

Basal cell carcinoma (BCC) is the most common skin cancer worldwide and its incidence is still rising with almost 10% each year worldwide [1, 2], thus representing a growing public health problem associated with negative psychosocial and economic consequences [3, 4].

These tumors that develop* de novo* have relatively uniform histology, and while not lethal they are locally invasive causing disfigurement and increasing morbidity due to frequent facial localization. Early diagnosis and prompt management are of crucial importance in order to prevent local tissue destruction or the occurrence of advanced disease. Although histopathological examination is considered the gold standard of diagnosis for BCC and other skin tumors, noninvasive and minimal invasive diagnostic tools have gained increased attention, as they do not imply performing a skin biopsy [5]. Among these novel optical imaging techniques, dermoscopy and reflectance confocal microscopy allow a rapid,* in vivo*, noninvasive micromorphological evaluation of skin tumors and combining these techniques can increase the diagnostic accuracy in different subtypes of BCC [6–8].

Known risk factors for developing BCCs include Fitzpatrick type I phototype, freckling and sunburns in childhood, family history of skin cancer, iatrogenic immunosuppression, and internal or external exposure to carcinogenic chemicals, especially arsenic [9] and high cumulative exposure to UV light [10]. Among these, UV radiation is considered by most as the main carcinogen [11] and around 80% of BCCs occur on sun-exposed areas, mostly the head and neck.

Regional anatomical differences, such as type and density of hair follicles, could explain why the back of the hands, despite extensive sun exposure, is a rare location for these tumors.

Carcinogenic processes [12, 13] are likely the result of multifarious interactions between host genome and environmental factors. Because of its morphological similarities to the undifferentiated epidermal basal cells, BCC provides an excellent model for identifying differentially expressed genes when compared to normal cells [14]. Genomic and proteomic techniques have both the potential to deliver new biomarkers [15]. However, gene transcript levels do not always correlate with protein expression due to transcriptional/translational control and high-throughput proteomic technologies are preferred for the measurement of proteins associated with pathological states [16]. Moreover, in early stages of the neoplastic process, individual proteomic platforms or platform combinations are used to characterize the great number of intact and cleaved proteins that can separate patients from healthy subjects [17]. Further, novel soluble and/or tissue-specific biomarkers can be developed for diagnosis, prognosis, and therapy monitoring in various malignancies [18–20], including BCC and other nonmelanoma skin cancers [21].

## 2. Sporadic Basal Cell Carcinoma

**Figure 1:**
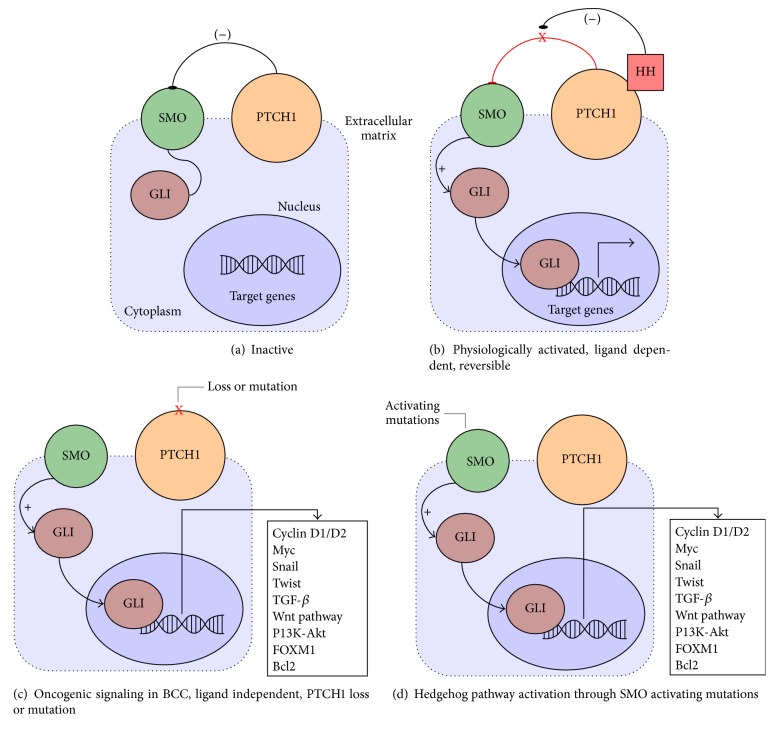
*Sonic hedgehog (SHH) signaling pathway*. (a) In the absence of the HH ligand, PTCH1 represses signaling through SMO causing GLI to remain inactive in the cytoplasm. (b) During physiological activation of the pathway the HH ligand binds to PTCH1, ending SMO suppression and causing activation and nucleus translocation of GLI thus influencing target genes expression. (c) Pathological activation of the SHH pathway through loss or mutational inactivation of PTCH1, suspending SMO inhibition in Gorlin-Goltz and sporadic BCCs (twist, FOXM1, Wnt pathway molecules, and others are viable biomarkers in BCC proteomic studies). (d) SMO activating mutations, found in sporadic BCCs, showing similar effects on the SHH pathway. HH:* sonic hedgehog* ligand; PTCH:* protein patched hedgehog* receptor, SMO: smoothened receptor, GLI: GLI factor, and Wnt:* wingless* signaling pathway.

Multiple signaling pathways are altered in carcinogenesis [22], and changes that lead to sonic hedgehog (SHH) patched 1 signaling pathway dysregulation have been recognized as essential events for sporadic BCC development [23] (see Figure 1). Gene expression alteration is an important event in tumor cells and mutations in the patched 1 gene are frequently present in BCCs [14].

Gli1 (glioma 1) transcription factor is an important downstream effector in the SHH pathway and has regularly been found upregulated in basal cell carcinomas [23].

GlI2 (glioma 2) is another key protein in BCC carcinogenesis and is also required for normal hair follicle development in the embryonic stages [24]. It has been proved that overexpression of GlI2 in the basal keratinocytes of transgenic mice leads to the development of multiple BCC-like tumors [25]. In addition, GLI2 has been regularly found upregulated in cancers presenting complex genomic alterations [26].

FOXM1, a Forkhead box protein, is a downstream molecule of SHH/Gli1 that has been found to be overexpressed in BCC and because of its role in cell proliferation it is thought to be one of the causes for aberrant SHH signaling in BCC tumorigenesis [14].

Lam et al. found another Forkhead box protein, FOXO3A, to be overexpressed in BCCs. FOXO3A, a transcription factor known for its involvement in cell-cycle arrest mediation, apoptosis, and DNA repair [27], also plays a key role in oxidative stress protection through upregulation of several antioxidants, such as SOD2 and catalase.

In a 2008 study Asplund et al. compared gene expressions of normal epidermal basal cells to those of BCC cells. The results revealed 201 upregulated and 160 downregulated genes in BCC cells compared to normal basal cells. Among them, they identified differentially expressed genes implicated in cell differentiation (aquaporin 3 and envoplakin), adhesion (claudin-1 and CD44), communication (desmoglein 2), and immune response (CD40 and MHC class II proteins). Consistent differences in immunoreactivity between tumor and control cells were found in half of gene products: claudin-1, cystatin A, CD44, calgranulin A (S100A8), prostaglandin-endoperoxide synthase 1 (COX-1), junctional adhesion molecule 3 (JAM3), envoplakin, and c-myc. Several members of the Wnt pathway were also found to be upregulated: Wnt receptor frizzled 8, *β*-catenin, Lef-1, and basonuclin-2. Desmoglein 2, a desmosomal cadherin strongly expressed in the bulge area and basal layer of the outer root sheath [28] and weakly in interfollicular epidermis [29], was also found to be overexpressed in BCC tumor cells. Several proteins were downregulated, namely, CD40, MHC class II molecules, Fc fragment of IgG binding protein, and immunoglobulin superfamily member 1 [14].

Zali [30] found that C3b, which has important roles in opsonisation and activation of the alternate pathway [31], had elevated expression levels in BCC cells when compared to controls, while transthyretin and ceruloplasmin had lower expressions. These results are supported by a previous study that found ceruloplasmin significantly decreased in BCC patients compared to healthy controls [32], probably owing to prolonged exposure to ultraviolet radiation. As no expression for aldolase C, FGG (Fibrinogen gamma chain), Prx-cis (periaxin), prothrombin, VDAC (voltage-dependent anion channels), and LRG (leucine-rich alpha-glycoprotein) was found in tumor cells, they were considered negative markers for BCC. In addition, the author underlines the necessity for further studies of these biomarkers in mucous secretions and blood [30].

## 3. Recurrent and Subsequent Basal Cell Carcinomas

Although various risk factors for increased incidence of recurrent BCC exist [33], none of them should be considered individually. Clinical risk factors for BCC recurrence include male sex, lesion topography (centrofacial region involving the inner canthus, nostrils, and periauricular area), tumor size (recurrence rate increases by 7% for each millimeter of increase in tumor diameter), photosensitivity, and over 60 years of age at first presentation [34, 35]. Aggressive-growth morphologic variants such as sclerosing, mixed (nodular and sclerosing), and less commonly superficial multifocal type are considered by some [36, 37] to be histological risk factors.

A proteomic approach of potential predictive markers for BCC recurrence employed the study of cyclooxygenase-2, ezrin, and matrix metalloproteinase-9 [33].

One study found that overexpression of COX-2 plays a significant role in carcinogenesis through several mechanisms such as enhancement of cellular proliferation, promotion of angiogenesis, inhibition of apoptosis, stimulation of invasion, and suppression of immune responses [38]. The promotion of tumor cell proliferation appears to result from cooperation between COX-2 and various cellular signaling pathways [39, 40]. Moreover, a connection between COX-2 overexpression and increased levels of vascular endothelial growth factor-A (VEGF-A), CD31 positive vessels, and regulators of apoptosis Mcl-1 and Bcl2 has been implied by previous studies [41]. El-Khalawany and Abou-Bakr found that 90.9% of recurrent BCCs (rBCC) expressed COX-2 compared to only 59.1% of nonrecurrent BCCs (nrBCC) [33]. Conversly, it was shown that COX-2 inhibition can mitigate tumor growth, decrease the expression cell proliferation markers, and promote cancerous cell apoptosis [39, 40].

Ezrin is a cytoplasmic peripheral membrane protein belonging to the ERM (ezrin, Radixin, and Moesin) protein family and acts as an intermediate between the plasma membrane and the actin cytoskeleton having a significant role in cell surface structure adhesion, migration, organization, tumor growth regulation and progression, and metastatic spread of numerous cancers [42, 43]. Higher frequencies for ezrin immunopositivity have been observed in rBCCs than in nrBCCs [33]. While Bagheri and collaborators found ezrin expression in 93% of the tested BCCs, no significant difference regarding ezrin expression was observed between different BCC subtypes. The authors did note a difference between the intensity of staining between BCC subtypes, with morpheaform BCC having a significantly higher intensity of staining compared to the nodular variant [44]. Some studies reported a higher ezrin expression in SCC compared with less aggressive tumors such as solar keratosis, keratoacanthoma, or Bowen's disease [42]. Moreover, in primary cutaneous melanomas ezrin expression was found to be associated with depth of invasion, progression, and metastasis [45]. These results are supported by a previous study [46] that found 92% positivity for ezrin expression in 25 BCCs. The authors, however, did not find any difference regarding positivity or staining pattern between BCCs and SCCs. They hypothesized that the intensity rather than the pattern of ezrin expression had a more probable impact on the tumor behavior but admitted that larger studies are needed for clarification.

Matrix metallopeptidase 9 (MMP-9), an enzyme also known as 92 kDa type IV collagenase, is a matrixin encoded by MMP-9 gene. Along with other molecules, it plays an important role in neutrophil migration across the basement membrane [47], angiogenesis, and neovascularization in tumoral tissues through recruitment of endothelial stem cells [48] and wound repair by stimulation collagen contraction [49]. MMP-9 was detected by ISH (*in situ* hybridization) in the stromal fibroblasts adjacent to tumor invasion sites in infiltrating basal cell and squamous cell carcinomas and in the eosinophils infiltrating the dermis in response to invasive BCC [50, 51] and another study found an increased expression of MMP-9 and MMP-2 in SCC versus BCC [52]. Dumas et al. considered the reduced expression of collagen IV accompanied by the increased expression of MMP-9 and MMP-2 could explain the increased aggressive behavior of SCC over BCC [52]. One study [33] did not find any statistically significant difference between MMP-9 expressions in recurrent versus nonrecurrent BCCs.

In a prospective study, Glaser et al. [53] measured the levels of mRNA for CD3e (a T-cell surface marker), CD25 (alpha chain of IL-2 receptor expressed on activated T-cells and B-cells), CD68 (marker for monocytes/macrophages), the cell surface glycoprotein ICAM-1 (intercellular adhesion molecule-1), and the cytokines interferon-gamma (IFN-*γ*) and IL-10 in BCC tumors from 138 patients. The median follow-up in this study was 26.6 months. It was revealed that subjects with initially low CD3e, CD25, CD68, and ICAM-1 mRNA levels had a significantly shorter tumor-free period (*p* = 0.03, *p* = 0.02, *p* = 0.003, and *p* = 0.08, resp.). It was also observed that nodular morphologies had lower gene expression levels compared to superficial or mixed tumors. The authors could not link IFN-*γ* mRNA levels to the risk of subsequent tumors [53]. This information shows that immune cell related gene expression in an initial BCC tumor could be used to predict subsequent BCC development. These results have been confirmed by other studies [54] which found elevated mRNA levels of IFN-*γ*, IL-2, and CD3e in regressing BCCs.

Hunt et al. conducted a study of primary BCCs with and without histological evidence of regression, proposing that some tumors induced immune responses capable of tumor disruption. They reported a significant increase in CD3 in tumors that present active regression compared to those showing no regression. They also found that expression of CD25 was greater in actively regressing BCCs compared to tumors that had no current or past regression [55].

These findings reinforce the importance of inflammatory and immune cells in tumor progression mediation.

## 4. Aggressive, Metastatic, and Giant Basal Cell Carcinoma

Numerous studies have employed proteomics in their attempt to characterize aggressive BCC and distinguish it from nonaggressive variants.

Ansarin et al. [56] found that elevated p53 protein expression could be considered a predictor of BCC aggressive behavior. However, another study [57] reported that even though differences in* p53* gene mutation frequency, types of mutations, and hot spots between aggressive and nonaggressive BCC exist, they do not clearly predict tumor behavior. Yu et al. [58], in a 2008 gene expression study, found that nodular and superficial BCCs demonstrate similar transcriptional profiles, but different from the morpheaform subtype, which shows a more diverse gene expression pattern, reflecting its invasive nature. However, Howell et al. [59] could not distinguish nodular from sclerosing BCC subtypes by their gene expression patterns.

As a common trait to all epithelial-derived tumors, BCC can express transcription factors like Snail and Twist 1 or mesenchymal markers like the cell adhesion molecule N-cadherin.

The basic helix-loop-helix (bHLH) transcription factor Twist 1 was initially identified in an experimental tumor model as a major regulator of epithelial to mesenchymal transition (EMT) [60]. It was also found to be significantly upregulated in patients with metastatic breast cancer when compared to early disease stages [61].

Epithelial to mesenchymal transition (EMT) is a complex process by which cells lose their epithelial traits and gain a mesenchymal-like phenotype. Numerous factors, such as transforming growth factor beta (TGF-*β*), epidermal growth factor (EGF), and Wnt-b signaling, have been described to promote the expression of transcription factors Twist 1 and Snail in epithelial cells, resulting in decreased expression of E-cadherin, upregulation of N-cadherin, vimentin, and fibronectin and the acquisition of morphological and functional characteristics of mesenchymal tissue cells [62].

In 2012, Majima et al. [63] present a case of morphoeic and multiple organ metastatic BCC exhibiting induction of Twist 1 and epithelial to mesenchymal conversion of cadherins in a 51-year-old Japanese male. Twist 1 expression was analyzed by immunohistochemistry on formalin-fixed paraffin-embedded sections of the tumor with representative sections from nodular BCCs serving as controls. Cells at the invasive front of the primary tumor proved to be positive for Twist 1, whilst cells from the tumor center were negative for this marker. Control cells from nonmetastatic nodular BCCs did not show nuclear Twist 1 expression. Additionally, cadherins (E-cadherin and N-cadherin) were assessed by double immunofluorescence stains that showed strongly expressed E-cadherin in nonmetastatic nodular BCC and very low expression of this epithelial marker in cells from the metastatic BCC. By comparison, while no N-cadherin expression could be detected in the control tumors, cells from the metastatic BCC were found to express N-cadherin at the invasive front. Cells from a metastasis showed high expression levels of Twist 1 and N-cadherin and notably decreased expression of E-cadherin. It has been previously shown that E-cadherin, a calcium-dependent cell adhesion molecule, plays a crucial role in tumor invasion suppression, and its loss of function is associated with increased tumor aggressiveness [64]. Pizarro reported reduced E-cadherin expression in infiltrative BCCs [65] that have also been previously shown to produce MMP-7 [66]. The authors consider Twist 1 a viable biomarker for either highly invasive or metastatic BCC [63]. Sasaki and collaborators found that in ESCC (esophageal squamous cell carcinoma) Twist or E-cadherin expression was correlated with tumor attributes, such as tumor stage, depth of invasion, lymphatic invasion, and regional and distant node metastasis, making their evaluation useful for determining prognosis [67].

Maspin, a protease inhibitor and a member of the serpin family, is the product of a tumor suppressor gene with an active role in apoptosis and inhibition of tumor invasion, metastasis, and angiogenesis [68]. Whereas Bagheri et al. [44] report expression of maspin in 74.4% of their samples of BCCs, they could not find a significant difference between BCC subtypes concerning maspin expression and intensity of staining. These results are in accordance with another study [69] in which maspin expression in BCC was found to be 87.5%. Although biased by the inclusion of 3 metatypical BCCs, Abdou et el. report a maspin expression in BCC of only 48% [70].

Rates for metastatic BCC are around the 0.55% mark, making it a very rare occurrence [71]. Defined by the AJCC (American Joint Committee on Cancer) as a tumor larger than 5 cm in diameter, giant BCC is also a very rare variant [72].

Alpha-smooth muscle (*α*-SMA) actin, an isoform typical of smooth muscle cells (SMC) encoded by the ACTA2 gene on chromosome 10q22-q24, is present in the skin in the arrector pili muscles, fibroblasts surrounding anagen hair follicles, myoepithelial cells of eccrine glands, perivascular pericytes, and vascular smooth muscle [73]. In several studies [74, 75] the expression of *α*-SMA was significantly higher in the stroma of aggressive BCCs when compared to nonaggressive BCCs. Adegboyega et al. implied that stromal expression of *α*-SMA was in fact restricted to aggressive tumors at the same time being highly predictive of aggressive behavior [74].

Recently, Motegi et al. studied the expression of Twist 1 and *α*-SMA in the stromal cells of a metastatic giant basal cell carcinoma. They concluded that although Twist 1 induced EMT of tumor cells might have been linked to distant organ metastases in their case, the presence of *α*-SMA in myofibroblasts surrounding BCC tumoral cell nests could certainly represent one of the trademarks of BCC aggressiveness [76].

Oh et al. suggested that membrane type-1 matrix metalloproteinase (MT1-MMP) and *β*-catenin could be considered biomarkers for high-risk BCC due to their important role in locally invasive and destructive growth tumor behaviour [77]. El-Bahrawy et al. noted that *β*-catenin is found mainly in the membrane of tumor cells of high-risk BCC and suggested that a molecular mechanism, other than the aberrant E-cadherin/catenin complex, is involved in these high-risk subtypes of BCC [78].

ER homeostasis in tumoral cells is dysregulated by either physiological or pathological stimuli, such as oxidative stress, DNA-damage, nutrient deficiency, calcium-depletion, certain growth factors, and oncogenic factors. Under such conditions, unfolded and misfolded proteins accumulate, leading to ER stress and the activation of ER-specific signaling pathways [79].

Endoplasmic reticulum protein 29 (ERp29) is a chaperone protein found in the lumen of the endoplasmic reticulum (ER) [80]. It is thought to play a role in protein processing and transport in the early secretory pathway. Erp29 is expressed at varying levels practically in every tissue, yet its precise role in the pathogenesis of neoplasia remains unknown [81].

One study pointed out the tumor suppressive role of ERp29 demonstrated by inhibition of tumor formation in mice xenografts. The authors also suggested that overexpression of ERp29 could indirectly result in activation of genes with tumor suppressive functions, like E-cadherin and spleen tyrosine kinase [82].

Cheretis et al. studied the implication of ERp29 in the pathogenesis of cutaneous BCC. The results revealed that 37.5% of all analyzed tumors expressed ERp29. Infiltrating carcinomas displayed more intense immunoreactivity compared to superficial variants which displayed less intense anti-ERp29 staining [81]. According to this study ERp29 is expressed in a subset of BCC in which the infiltrating morphologies exhibit the highest incidence of immunopositivity.

Following cell metastasis, disseminated mesenchymal cancer cells can be reversibly converted to an epithelial cell state by mesenchymal-epithelial transition (MET) [83]. Considering that ERp29 can drive MET in mesenchymal breast cancer cells [82], ERp29 may play an important role in promoting distant metastasis during disease progression in basal cell carcinoma.

## 5. Nevoid Basal Cell Carcinoma Syndrome (Gorlin-Goltz Syndrome)

The molecular mechanisms underlying the pathogenesis of multiple BCCs in Gorlin-Goltz syndrome (GS) differ considerably from those of sporadic BCC development. Patients suffering from GS develop multiple BCCs at young ages and tumors are more often localized on non-UV-exposed skin [84]. Genetic anomalies of the PTCH1 gene in GS include nonsense, frameshift, in-frame, splice-site, interstitial, and missense mutations [85]. Immunohistochemical analysis with nonmutated site-targeting anti-PTCH1 antibody cannot differentiate GS associated from sporadic forms of BCC, because both show comparable patterns and intensity of staining [86].

A comparison of whole genome expression [87] between GS and healthy controls revealed a genomic signature which included several genes with known associations with tumor growth and invasiveness. The authors also report that the genotype of PTCH1+ fibroblasts from tumor-free skin of NBCCS patients was similar to that of BCC associated fibroblasts to the extent that NBCCS fibroblasts overexpressed mRNAs encoding MMP-1, MMP-3, and tenascin C, proproliferative factors such as fibroblast growth factor 7 (FGF-7) and stromal cell-derived factor 1 alpha. In addition, there was strong MMP-1 overexpression in PTCH1+ fibroblasts obtained from NBCCS patient compared to healthy donors [87].

Ponti et al. [88] analyzed profiles of fibroblast conditioned culture media of PTCH1+ and compared them to nonmutated fibroblasts. Statistically significant differences between two different types (missense versus nonsense) of PTCH1 mutations in the profiles of fibroblasts from conditioned media were revealed. Results confirmed previously documented [87] MMP-1 overexpression in PTCH1 mutated fibroblasts, thus confirming the relationship between PTCH1 mutation and MMP-1-related neoplastic transformation of epithelial cells.

Adding to these findings, matrix metalloproteinase-1 (MMP-1) was identified by Weiss et al. to be a downstream target of Twist 1. According to their study, MMP-1 may also enhance cellular motility and invasion of BCC by disrupting the basement membrane and degrading the stromal matrix [89].

MMP-1 can thus be considered a novel marker for target therapy in the context of NBCSS. Future studies of this particular proteomic signature could prove useful for the clinical, therapeutic, and prognostic evaluation of these patients.

## 6. Conclusions

In summary, it is apparent that gene expression alteration induced by different pathways in tumor cells due to the variation in the expression of other factors is an essential event in BCC carcinogenesis. Analyzing gene expression and proteomic profiles of tumor cells and its microenvironment in different tissue or fluid biological samples indicates new candidate molecules involved in skin cancer pathogenic pathways that might represent future predictive and prognostic biomarkers in BCC and other skin cancers.
